# Retraction: Long non-coding RNA XIST promotes proliferation, autophagy and inhibits apoptosis by regulating microRNA-30c/ATG5 axis in gastric cancer

**DOI:** 10.1039/d1ra90008k

**Published:** 2021-01-20

**Authors:** Laura Fisher

**Affiliations:** Royal Society of Chemistry, Thomas Graham House Science Park, Milton Road Cambridge CB4 0WF UK advances-rsc@rsc.org

## Abstract

Retraction of ‘Long non-coding RNA XIST promotes proliferation, autophagy and inhibits apoptosis by regulating microRNA-30c/ATG5 axis in gastric cancer’ by Mingjian Liu *et al.*, *RSC Adv.*, 2018, **8**, 37508–37517, DOI: 10.1039/C8RA07852A.

The Royal Society of Chemistry hereby wholly retracts this *RSC Advances* article due to concerns with the reliability of the data. The images in the article were screened by an image integrity expert who found that the two sets of western blots in Fig. 2 are identical but represent different experiments. Furthermore, the western blots and many other features of the article were found to be unexpectedly similar to western blots and features in a number of other papers with no overlapping authors.

The authors were asked to provide the raw data for this article, but did not respond. Given the significance of the concerns about the validity of the data, and the lack of raw data, the findings presented in this paper are not reliable.

The authors have been informed but have not responded to any correspondence regarding the retraction.

Signed: Laura Fisher, Executive Editor, *RSC Advances*

Date: 7^th^ January 2021

## Supplementary Material

